# Chylous Ascites Secondary to Retroperitoneal Para-Aortic Lymphadenectomy: A Case Report

**DOI:** 10.7759/cureus.22560

**Published:** 2022-02-24

**Authors:** Yolanda Gil González, María Laseca-Modrego, Octavio Arencibia-Sánchez, Daniel González García-Cano, Alicia Inmaculada Martin Martinez

**Affiliations:** 1 Obstetrics and Gynecology, Complejo Hospitalario Universitario (C.H.U.) Insular-Materno Infantil, Las Palmas de GC, ESP; 2 Gynecologic Oncology, Complejo Hospitalario Universitario (C.H.U.) Insular-Materno Infantil, Las Palmas de GC, ESP; 3 Obstetric and Gynecology, Universidad Las Palmas de Gran Canaria, Las Palmas de GC, ESP

**Keywords:** oncology, surgery, cervical cancer, retroperitoneal para-aortic lymphadenectomy, chylous ascites

## Abstract

Chylous ascites is caused by an accumulation of lymphatic fluid in the peritoneal cavity secondary to a rupture or obstruction of the abdominal lymphatic ducts. It has a milky appearance and is rich in triglycerides. The most frequent etiologies are neoplasms, liver cirrhosis, and ruptured lymphatic vessels after abdominal surgery. Clinically, it manifests as abdominal distention and increased abdominal girth. The presence of triglycerides in ascites fluid is the most useful diagnostic criterion. Treatment consists of a high-protein diet with fat restriction and medium-chain triglyceride supplements. Surgery is reserved for refractory cases. We present the case of a 66-year-old patient with a diagnosis of chylous ascites secondary to retroperitoneal lymphadenectomy.

## Introduction

Chylous ascites is caused by an accumulation of lymphatic fluid in the peritoneal cavity [1]. It is a collection of chyle with a milky appearance, rich in triglycerides produced by the presence of lymph in the abdominal cavity. The most frequent etiologies are neoplastic processes, liver cirrhosis, and ruptured lymphatic vessels after abdominal surgery. It manifests as abdominal distention and the presence of abdominal ascites fluid. The presence of triglyceride concentrations above 200 mg/dl in the ascitic fluid analysis confirms the diagnosis [2-4]. According to the literature consulted [5-7], it is a rare finding with an incidence of 1/11,500-80,000 patients. 

There are a series of cases described, although no large clinical series have been published. The objective of the present case is to describe the report and carry out a review of the current bibliography on the management of chylous ascites.

## Case presentation

We present a 66-year-old woman diagnosed with locally advanced cervical cancer, FIGO IIB stage (2018), undergoing follow-up by the Gynecologic Oncology Unit. After the presentation to the tumor committee, it was decided to perform a staging retroperitoneal para-aortic lymphadenectomy before the radical treatment (concomitant chemotherapy and radiotherapy). Following the recommendations of the tumor committee, a laparoscopic retroperitoneal para-aortic lymphadenectomy was carried out without incident, with negative results for malignancy of all nodes removed. The immediate postoperative period was uneventful, with hospital discharge at 48 hours.

**Figure 1:**
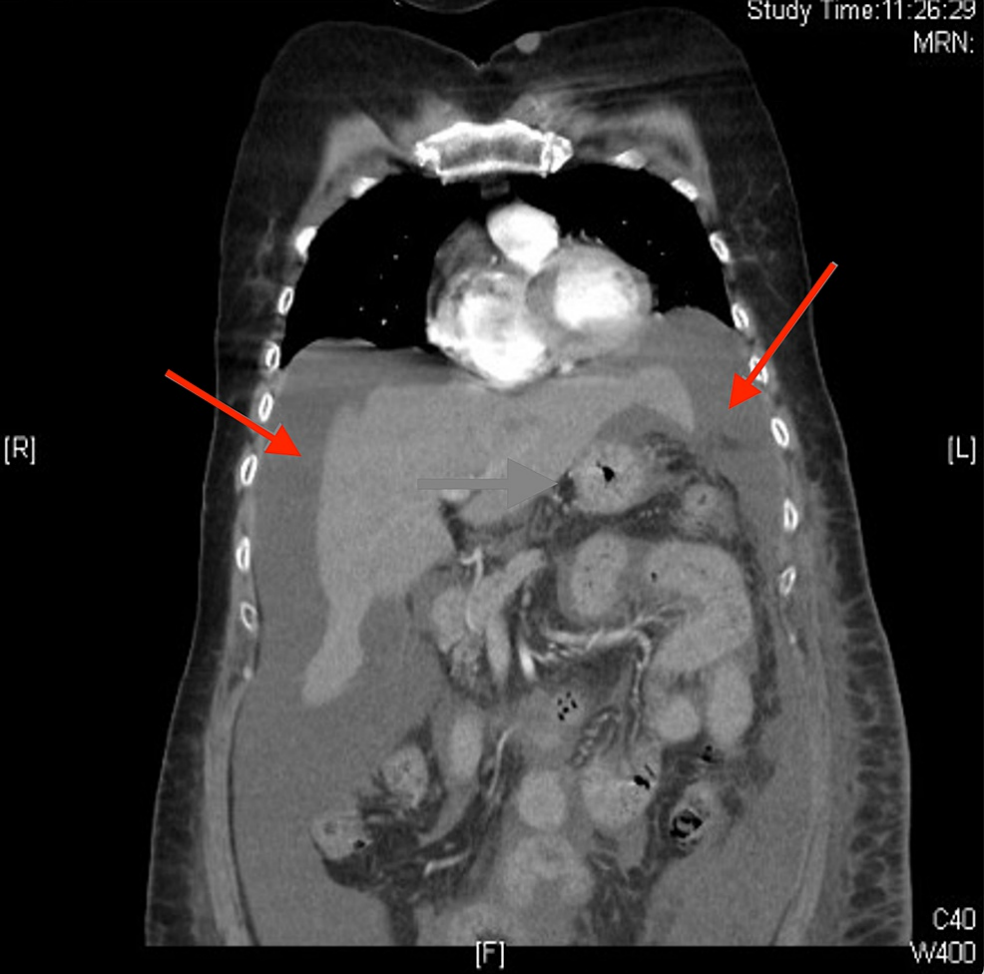
Coronal CT section showing abundant perihepatic, perisplenic and around bowel loops fluid. Red arrows point perihepatic, perisplenic and around bowel loops fluid.

**Figure 2:**
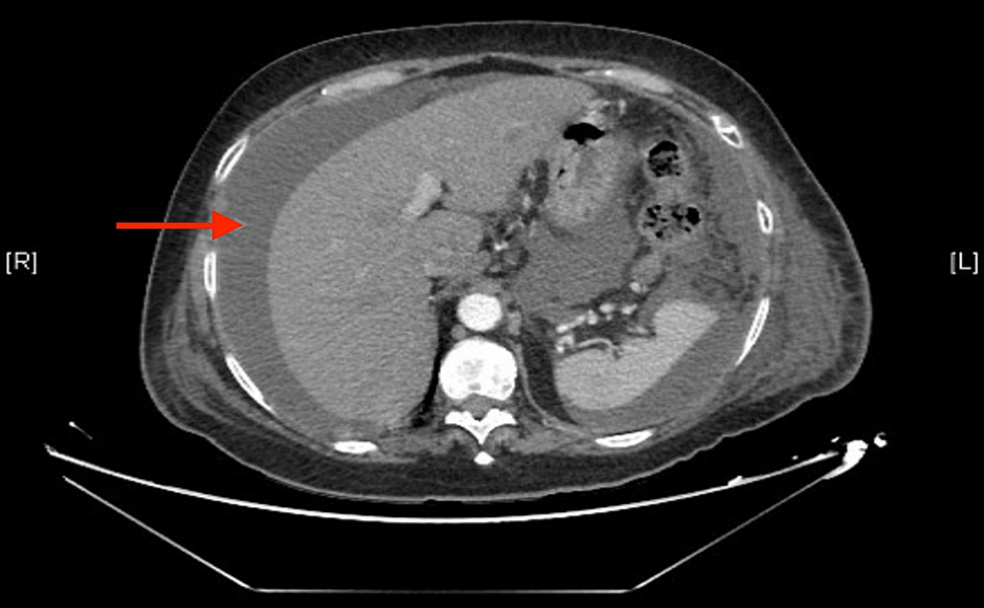
Sagital CT section showing abundant perihepatic, perisplenic and around bowel loops fluid. Red arrow point perihepatic, perisplenic and around bowel loops fluid.

One month after the surgery, the patient came to the emergency room with generalized abdominal pain, distension, diarrhea, early satiety, and increased girth that had evolved over a 48-hour period. On examination, she presented with visible abdominal distension and a positive ascites surge. A thoracic-abdomino-pelvic CT scan (Figures 1, 2) was performed as a complementary test. It showed abundant perihepatic, perisplenic and around bowel loops fluid, and a collection compatible with a retroperitoneal lymphocele, with no other pathological findings.

**Figure 3:**
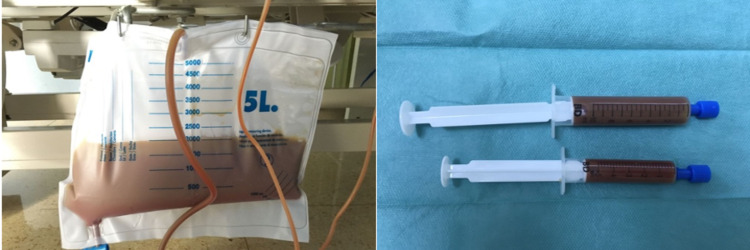
Ascitic fluid sample after paracentesis. It can be seen bloody milk-like ascites fluid

During her stay, an evacuating paracentesis was performed with the extraction of 5500 cc of bloody milk-like ascites fluid (Figure 3), with the improvement of symptoms. Given the suspicion of chylous ascites secondary to previous surgery, a sample of the fluid was sent for analysis, which revealed a triglyceride value of 357 mg/dl.

The patient was diagnosed with chylous ascites secondary to ruptured lymphatic ducts that had occurred as a result of the previously performed para-Aortic lymphadenectomy. She was treated with a high-protein diet with fat restriction, medium-chain triglyceride supplements at 20 ml/day, and subcutaneous octreotic 100 µg every eight hours. The evolution was satisfactory. She was discharged after four days. She continued the treatment as an outpatient. Octreotide was discontinued one month after the diagnosis, and the evolution was favorable. Subsequently, the patient received external radiotherapy, brachytherapy, and sensitizing chemotherapy as treatment for cervical cancer, presenting no complications. Twelve months after discharge, the patient remains on a low-fat, high-protein diet, supplemented with medium-chain triglycerides, and is making good progress.

## Discussion

In our center, over a period of 13 years, a total of 239 staging para-Aortic lymphadenectomies have been performed on locally advanced cervical cancer patients, with only one case of chylous ascites during this entire period, which represents 0.42% of the sample.

Chylous ascites is the accumulation of lymphatic fluid in the peritoneal cavity [1]. Its milky appearance is an expression of the high level of triglycerides (greater than 200 mg/dL [2-4]), especially chylomicrons [5]. In our case, after analyzing the sample, a triglyceride value of 357 mg/dl was obtained, which confirmed the diagnosis. It is a rare entity whose incidence varies depending on the literature that we consulted: 1 case for every 11,500 to 80,000 patients [5-7]. Its pathophysiology is explained as a disruption of the lymphatic system due to a traumatic injury or an obstruction of lymphovenous or lympho-lymphatic communications [6]. Fifty to ninety percent of lymphatic flow derives from the intestine and liver, and contains fat in the form of chylomicrons [5,8]. The etiology is divided according to atraumatic and traumatic causes. Traumatic causes are the most frequent (Table 1).

**Table 1:** Causes of chylous ascites.

Traumatic causes	Post-surgery: abdominal aorta resection or inferior vena cava, retroperiotoneal lymphadenectomy, vagotomy, nephrectomy, Nissen fundoplication, liver transplantation, etc. Direct trauma
Atraumatic causes	Neoplasia (stomach, esophagus, pancreas, endometrium, prostate, leukemia, sarcoma, neuroendocrine tumor, etc.), hepatic cirrhosis. Congenital causes: idiopathic, intestinal lymphangiectasia, primary lymphatic hypoplasia, lymphangiomatosis Inflammatory. Secondary to treatment with radiotherapy, pancreatitis, celiac disease, retroperitoneal fibrosis, sarcoidosis, constrictive pericarditis, Whipple's disease. Infectious: tuberculosis, filariasis, peritoneal dialysis, systemic lupus erythematosus, hyperthyroidism, congestive heart failure. Obstructive cause: adhesions, volvulus, aortic aneurysm, nephrotic syndrome.

The present case is a chylous ascites secondary to surgery. Its appearance varies according to the type of surgery, with interventions related to oncological processes, specifically retroperitoneal lymphadenectomy, the procedure with the highest risk [1].

Complications can be present early (during the first week) if there has been a rupture of the lymphatic vessels, or late (weeks or months) because of adhesions or extrinsic compression of the lymphatic vessels. This was most likely in our case due to the presence of a lymphocele, which could have caused the rupture of the lymphatic vessels.

Clinically, abdominal distention is the most common sign, and nonspecific abdominal pain, diarrhea, malnutrition, edema, early satiety, and dyspnea may also appear [3,4]. Weight gain can be observed due to fluid retention, or weight loss due to associated malnutrition. In the analysis, we could observe hypoproteinemia due to loss of albumin, fibrinogen and immunoglobulins, hyponatremia, metabolic acidosis, depletion of fat reserves, and fat-soluble vitamins, as well as lymphopenia. [1,8]. In the study of the ascitic fluid, the following could be observed: alkaline pH, high content of triglycerides and proteins, low cholesterol level, and a predominance of lymphocytes [9,10]. In our case, the triglyceride value was 357 mg/dl, which confirmed the diagnosis of chylous ascites. The most common characteristics of chylous ascites are shown in Table 2 [1,8-10].

**Table 2:** Characteristics of chylous ascites.

	Characteristics	Reference range
Color	Whitish, milky	
pH	Alkaline	>7
Triglycerides	Elevated	150-1100 mg/dl
Cholesterol	Decreased	<220 mg/dL
Total proteins	Elevated	>3 g/L
Cellularity	Lymphocyte	400-7.000 × 103/dL
LDH		110-200 Ul/L
Culture	+/-	
Cytology	+ in case of neoplasms	
Glucose	Decreased	<100 mg/dl

Treatment depends on the etiology. It should be as conservative as possible, and it will resolve in 67-75% of cases [1,6]. Depending on the severity, surgery may be indicated. Recommended treatments are as follows. (i) Diet (rich in protein and low in fat, supplemented with medium-chain triglycerides, prioritizing the oral route whenever possible) [5,6] and supplementation with fat-soluble vitamins to prevent the deficiency of essential fatty acids [11-13]. (ii) Somatostatin and octreotide (somatostatin analog); its action is not clearly known. It has been hypothesized that they act by reducing lymph excretion [6,13]. (iii) Other drugs: Diuretics (they facilitate the excretion of volume, reducing the formation of ascites), orlistat (a reversible inhibitor of gastric and pancreatic lipases that decreases enteral fat absorption [5,6,12]), ethylephrine (a sympathomimetic drug that works by contracting the smooth muscles of the thoracic duct and decreases chyle flow [14]). (iv) Surgery is recommended in cases where treatment is ineffective (persistence of more than two weeks), the appearance of important metabolic complications or debit is greater than 1000 cc/day for more than five days. In the latter cases, some authors argue that the response to conservative treatment is lower and surgery could be considered earlier [15,16]. Numerous techniques can be performed, such as ligation of the injured lymphatic duct, placement of a peritoneal venous shunt, percutaneous intrahepatic portosystemic shunt, and repeat paracentesis [9,11]. The prognosis is linked to the underlying cause, and it is worse in cases not related to postoperative complications [16].

## Conclusions

This is a rare case of chylous ascites secondary to retroperitoneal para-aortic lymphadenectomy that resolved with medical treatment. Chylous ascites secondary to postoperative complications can appear early, probably due to rupture of the lymphatic vessels, or late, as occurred in our case, due to adhesions or extrinsic compression of the lymphatic vessels (lymphocele). Treatment consists of a high protein diet with fat restriction and medium-chain triglyceride supplements, reserving surgery for situations refractory to treatment.
